# A Translational Quantitative Systems Pharmacology Model for CD3 Bispecific Molecules: Application to Quantify T Cell-Mediated Tumor Cell Killing by P-Cadherin LP DART^®^

**DOI:** 10.1208/s12248-019-0332-z

**Published:** 2019-05-22

**Authors:** Alison Betts, Nahor Haddish-Berhane, Dhaval K. Shah, Piet H. van der Graaf, Frank Barletta, Lindsay King, Tracey Clark, Cris Kamperschroer, Adam Root, Andrea Hooper, Xiaoying Chen

**Affiliations:** 1 Department of Biomedicine Design, Pfizer Inc., 610 Main Street, Cambridge, Massachusetts 02139 USA; 2 Division of Systems Biomedicine and Pharmacology, Leiden Academic Centre for Drug Research, 2300 RA Leiden, The Netherlands; 3 Janssen Research & Development, LLC, Spring House, Pennsylvania 19477 USA; 4 Department of Pharmaceutical Sciences, 455 Kapoor Hall, University at Buffalo, The State University of New York, Buffalo, New York 14214-8033 USA; 5 Oncology Research Unit, Pfizer Inc., 401 N Middletown Rd., Pearl River, New York 10965 USA; 6 Department of Biomedicine Design, Pfizer Inc., 1 Burtt Road, Andover, Massachusetts USA; 7 Established Med Business, Pfizer Inc., Eastern Point Rd, Groton, Connecticut 06340 USA; 8 Department of Immunotoxicology, Pfizer Inc., 558 Eastern Point Road, Groton, Connecticut 06340 USA; 9 Department of Clinical Pharmacology, Pfizer Inc., 10555 Science Center Dr., San Diego, California 92121 USA

**Keywords:** CD3 bispecific, translational modeling, quantitative systems pharmacology, PK/PD, immunotherapy

## Abstract

CD3 bispecific antibody constructs recruit cytolytic T cells to kill tumor cells, offering a potent approach to treat cancer. T cell activation is driven by the formation of a trimolecular complex (trimer) between drugs, T cells, and tumor cells, mimicking an immune synapse. A translational quantitative systems pharmacology (QSP) model is proposed for CD3 bispecific molecules capable of predicting trimer concentration and linking it to tumor cell killing. The model was used to quantify the pharmacokinetic (PK)/pharmacodynamic (PD) relationship of a CD3 bispecific targeting P-cadherin (PF-06671008). It describes the disposition of PF-06671008 in the central compartment and tumor in mouse xenograft models, including binding to target and T cells in the tumor to form the trimer. The model incorporates T cell distribution to the tumor, proliferation, and contraction. PK/PD parameters were estimated for PF-06671008 and a tumor stasis concentration (TSC) was calculated as an estimate of minimum efficacious trimer concentration. TSC values ranged from 0.0092 to 0.064 pM across mouse tumor models. The model was translated to the clinic and used to predict the disposition of PF-06671008 in patients, including the impact of binding to soluble P-cadherin. The predicted terminal half-life of PF-06671008 in the clinic was approximately 1 day, and P-cadherin expression and number of T cells in the tumor were shown to be sensitive parameters impacting clinical efficacy. A translational QSP model is presented for CD3 bispecific molecules, which integrates in silico, *in vitro* and *in vivo* data in a mechanistic framework, to quantify and predict efficacy across species.

**Electronic supplementary material:**

The online version of this article (10.1208/s12248-019-0332-z) contains supplementary material, which is available to authorized users.

## Introduction

Immunotherapy, which recruits a patient’s own immune system to kill cancer cells, has begun to revolutionize cancer treatment (1). Within the class of immune-oncology therapies are the bispecific immune cell re-targeting molecules (2). These are typically recombinant bispecific antibodies, or antibody fragments, with one binding domain targeting a specific tumor antigen of choice and the other domain targeting CD3 on T cells. Because CD3 serves as the signaling component of the T cell receptor (TCR) complex, these CD3 bispecific molecules enable T cells to circumvent the need for the interaction between the TCR and antigen presented by major histocompatibility complex (MHC) class I molecules. This expands the repertoire of T cells able to recognize the tumor and stimulate them to act as effector cells (3). Similar to the standard immune synapse formation, once a threshold of bispecific-mediated molecular interactions has been reached, CD3 signals the T cell to initiate a cytotoxic response toward the adjacent tumor cell expressing the specific antigen. Cytotoxicity is mediated by the release of cytotoxic granules containing perforin and granzymes by the T cell. Perforin is a pore-forming protein enabling entry of granzymes, and the granzymes trigger a caspase cascade that leads to apoptosis. Activation of T cells leads to the transient release of cytokines and T cell proliferation, recruitment, and infiltration into the tumor environment, which drives serial killing of tumor cells.

In 2014, blinatumomab (CD3-CD19) was the first CD3 bispecific construct approved in the USA for the treatment of resistant/refractory B cell acute lymphocytic leukemia (B-ALL) (4). Blinatumomab is also being investigated in phase 2 clinical trial in patients with resistant/refractory non-Hodgkin’s lymphoma (NHL) (5). The first generation bispecific T cell retargeting molecules such as blinatumomab are tandemly linked single-chain Fv (scFv) known as bispecific T cell engager (BiTE) molecules (2,3). These molecules are around 50 kDa and have a short circulating half-life (approx. 2 h) requiring constant infusion through the use of a pump to achieve a stable therapeutic exposure of the molecule (6). New generation CD3 bispecifics with a variety of formats are being tested in clinical trials. These include PF-06671008 which is a P-cadherin-specific LP DART: a molecule based on the DART^®^ platform, but containing a human IgG1 Fc domain to extend the half-life (7). This bispecific targets CD3 and P-cadherin expressed on solid tumors. P-cadherin is a member of a family of molecules that mediate calcium-dependent cell-cell adhesion and has been reported to correlate with increased tumor cell motility and invasiveness when over-expressed (8–10). Upregulation of P-cadherin has been reported in breast, gastric, endometrial, colorectal, and pancreatic carcinomas and correlates with poor survival of breast cancer patients (11–14). In contrast, P-cadherin has low expression in normal tissues, making it an attractive target for immunotherapy (12). In preclinical studies, *in vitro* and *in vivo* data indicate that PF-06671008 is a highly potent molecule eliciting P-cadherin expression-dependent cytotoxic T cell activity across a range of tumor indications (15). In addition, PF-06671008 is stable and has desirable biophysical and PK properties with a half-life of 3.7–6 days in mouse (7,15). PF-06671008 is currently being investigated in phase 1 clinical trials in patients with advanced solid tumors with the potential to have P-cadherin expression (https://clinicaltrials.gov/ct2/show/NCT02659631).

In order to characterize the *in vivo* efficacy of PF-06671008 in tumor-bearing mice, a quantitative systems pharmacology (QSP) model was established. This model integrates the PK of PF-06671008, its binding to shed P-cadherin and circulating T cells in the systemic circulation, its biodisposition in the tumor and the formation of a trimolecular complex (trimer) with T cells, and P-cadherin expressing tumor cells in the tumor microenvironment (TME). The model incorporates T cell kinetics in the tumor including T cell proliferation and contraction. The concentration of the trimer in the tumor is used to drive efficacy in mouse using an optimized transduction model of tumor cell growth and killing. In this manuscript, we discuss the use of the model to characterize the underlying pharmacology in mouse, and translation of the preclinical efficacy data to the clinic by incorporation of predicted human PK and disease parameters. The quantitative translational framework for CD3 bispecific molecules presented here can aid in drug design, candidate selection, and clinical dosing regimen projection.

## Materials and Methods

### *In Vivo* Studies

All procedures in animals were approved by the Pfizer Institutional Animal Care and Use Committees and studies were performed according to established guidelines.

### PF-06671008 Mouse PK Study

PF-06671008 was administered as a single intravenous (IV) dose of 0.05 or 0.5 mg/kg to HCT-116 tumor-bearing female NOD-scid IL-2rg^null^ (NSG) mice, (*n* = 3/time point/dose) with or without human peripheral blood mononuclear cell (PBMC) engraftment. Mice were injected with 5 × 10^6^ HCT-116 cells in Matrigel subcutaneously in the dorsal left flank. When the tumors had grown to approximately 0.5 g in size (after 14 days), the mice were administered PF-06671008. Serum and tumor samples were collected at predetermined time points from 5 min to 240 h post-dose.

### ELISA Assay to Quantify PF-06671008

PF-06671008 concentrations in mouse serum and tumor homogenate were determined using an enzyme-linked immunosorbant assay (96-well format) with colorimetric detection. Briefly, the capture protein was a polyclonal goat antibody recognizing the CD3 scFv domain and the detection antibody was a goat anti-human IgG-Biotin (Qualex), followed by HRP-streptavidin conjugate (Jackson ImmunoResearch, West Grove, PA). Optical density was measured on a spectrophotometer (molecular devices). The lower limit of quantitation (LLOQ) of the assay was 12.5 ng/mL for serum samples and 1.5 ng/mL for tumor samples. The minimal required dilution was 1:25 for serum and 1:6 for tumor.

### Flow Cytometric Tumor-Infiltrating Lymphocyte Analysis

HCT-116 tumor-bearing mice (*n* = 3) engrafted with human PBMC and administered a single IV dose of 0.01, 0.05, or 0.5 mg/kg PF-06671008 were euthanized pre-dose and 24, 72, and 144 h following dosing to assess tumor-infiltrating human CD3+ lymphocytes. Tumor samples were collected into gentleMACS C tubes containing human tumor cell dissociation buffer (Miltenyi Biotech) and processed to single-cell suspensions using the manufacturer’s suggested protocol for soft human tumors using the gentleMACS tissue dissociator (Miltenyi Biotech). After subsequent washing steps and live cell counting (using a hemocytometer and trypan blue exclusion), 1 × 10^6^ live cells from each sample were collected and stained with CD3 FITC (BD Pharmingen) for 30 min on ice. Samples were analyzed using LSRII with FACS Diva software (BD Pharmingen). Absolute numbers of CD3+ T cells per gram of tumor were then calculated using the number of CD3+ events and sample tumor weight.

### PF-06671008 Mouse Xenograft Studies

Mouse xenograft studies were completed in human T cell engrafted (HCT-116) or adoptive transfer (HCT-116 or SUM-149) established tumor models. In the human T cell engrafted model, tumor cells (5 × 10^6^ HCT-116) were implanted subcutaneously (SC) into the right flank of 6–8-week-old female NSG mice as a 0.2 mL bolus mixed with 4 mg/mL Cultrex basement membrane extract (Trevigen) in PBS. Seven days prior to randomization, mice were inoculated with 5 × 10^6^ or 2.5 × 10^6^ freshly isolated human PBMC as an intraperitoneal injection of 0.2 mL cell suspension in PBS. In addition to vehicle (PBS), dose levels of 0.01, 0.05, 0.1, 0.15, and 0.5 mg/kg PF-06671008 were administered for HCT-116 studies (*n* = 10/dose). The doses were administered IV as a q7d × 2 regimen.

For the T cell adoptive transfer established tumor model, 8- to 10-week-old NSG mice were inoculated with either 5 × 10^6^ HCT-116 cells in the flank or 5 × 10^6^ SUM-149 cells in the mammary fat pad in a total injection volume of 0.2 mL, 7 days prior to randomization. HCT-116 cells were suspended in PBS, while SUM-149 cells were suspended in growth media and mixed 1:1 with Matrigel basement membrane matrix (BD Biosciences, San Jose, CA). T cells, which had been isolated from PBMCs, were activated and expanded using Dynabeads Human T-Expander CD3/CD28 magnetic beads (Life Technologies) for 6–9 days, depending on the study, were harvested, and resuspended in PBS at 1 × 10^7^ cells/mL for *in vivo* inoculation. An initial dose of PF-06671008 or vehicle was administered to mice on day 0 and on the following day, mice were inoculated with 0.5, 1, 2, 2.5, or 5 × 10^6^ T cells/mice IV. In addition to vehicle, dose levels of 0.05, 0.15, and 0.5 mg/kg PF-06671008 were administered for HCT116 xenograft studies and 0.05, 0.15, and 0.5 mg/kg PF-06671008 for SUM149 xenograft studies (*n* = 10/dose). The doses were administered IV as a q7d × 3 or q7d × 5 regimen.

Tumor volume was measured using a digital Vernier caliper (Mitutoyo America, Aurora, IL), and volumes were calculated by the use of the modified ellipsoid formula ½ (width^2^ × length). Tumor measurements were collected twice weekly, with continuous health monitoring, until the animals had to be euthanized due to tumor burden or health concerns out to a maximum of 16 days (HCT-116 in the human T cell engrafted model), 42 days (SUM-149 adoptive transfer), or 65 days (HCT-116 adoptive transfer). For a full description of the mouse xenograft studies, please refer to reference (15).

### PF-06671008 Cynomolgus Monkey PK Study

The PK of PF-06671008 in cynomolgus monkey was evaluated following IV bolus and SC administration at weekly escalating doses for 1 month. The IV doses administered were 1.1/3.3, 3.3/10, or 10/20 μg/kg/week, and the SC dose administered was 10/30 μg/kg/week. This study has been described previously (16).

### Measurement of Soluble P-Cadherin

Baseline sPcad levels were measured in cynomolgus monkey, healthy volunteer, and cancer patient serum samples (Bioreclamation). sPcad levels were also measured in cynomolgus monkey after treatment with PF-06671008 (in-house samples). A qualified Meso Scale Discovery (MSD) human P-cadherin kit was used to measure soluble P-cadherin levels, as described previously (16).

### Modeling of Mouse Tumor Growth Inhibition Data

**Fig. 1:**
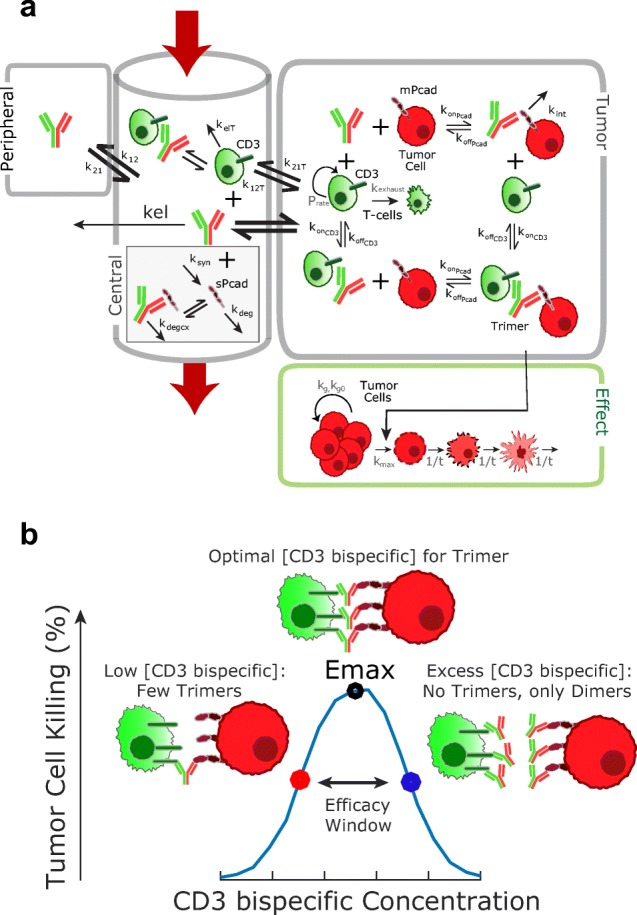
**a** Translational quantitative systems pharmacology model for CD3 bispecific molecules. Parameter descriptions and values are summarized in Tables I to III. The figure represents both mouse and human models, with the following exceptions: binding to sPcad was only included in the human model and T cell proliferation/ exhaustion in the tumor were only included in the mouse model. **b** Schematic of the bell-shaped concentration relationship which can be observed for CD3 bispecific molecules. Formation of trimers between drugs, T cells, and tumor cells, is required for efficacy. The QSP model predicts trimer concentration and links it to tumor cell killing

A QSP model was constructed to describe the disposition of PF-06671008 and T cells in the central compartment and tumor of the xenograft mouse models (Fig. 1a). The model accounts for the binding of PF-06671008 to tumor cells and T cells in the extracellular space of the TME to form trimers. The trimers are assumed to drive tumor cell killing. Description of all the symbols and parameters used in the mouse equations are shown in Tables I and II.

1. Modeling of PF-06671008 and T cells in central/peripheral compartments and distribution to the tumor: Following systemic administration to mouse, PF-06671008 is assumed to be able to distribute to a peripheral compartment, distribute to the tumor, bind to circulating T cells, or be cleared from the central compartment. In the mouse model, PF-06671008 does not bind to sPcad.

- Mouse PK: The mouse serum concentration profiles in human PBMC-engrafted mice, following IV administration of PF-06671008 at 0.05 and 0.5 mg/kg, were described using a two-compartment model with linear elimination from the central compartment (Eqs. 1 and 2). *C1*, *C2*, *and C3* are the concentrations of the drug, PF-06671008, in plasma, peripheral compartment, and tumor, respectively. *kel* is the elimination rate of PF-06671008 from the central compartment. *k12* and *k21* are the inter-compartmental rate constants describing the distribution of PF-06671008 between the central compartment and the peripheral compartment. These values were fixed in the subsequent TGI modeling. Distribution of free PF-06671008 to the extracellular environment of the tumor was characterized using tumor disposition equations (Eqs. 3 and 4) that have been described previously (19,25,26). Briefly, *P* is the rate of permeability and *D* is the diffusion of the drug, across and around the tumor blood vessels. *R*_*cap*_ is the radius of the tumor blood capillary, *R*_*krogh*_ is the average distance between 2 capillaries, *R*_*tumor*_ is the radius of the tumor, and *ε* is the tumor void volume for the drug.
- Binding to T cells: Binding of PF-06671008 to circulating T cells was determined from CD3 binding (*k*_*onCD3*_ and *k*_*offCD3*_), the number of CD3 receptors per T cell (*CD3*) and number of T cells in the central compartment, or plasma (*Tcells*_*p*_). These values were used to calculate total CD3 in the central compartment (*TotCD3*_*p*_) (Eq. 5). Binding to CD3 (and P-cadherin) was determined using surface plasmon resonance (SPR) assays run on a Biacore instrument as described previously (7). The number of CD3 receptors per T cell was taken from literature data (17,18). The number of T cells administered per mouse was used to inform the initial number of T cells in the central compartment. See Eq. 6 for binding of PF-06671008 to CD3 in the central compartment. *DCD3p* and *DCD3t* are the concentration of drug-CD3 dimers in plasma and tumor, respectively.
- T cell trafficking: Following administration of T cells to the mouse, T cells were assumed to be able to distribute to the tumor (Eqs. 7 and 8), bind to PF-06671008, or be cleared from the central compartment. *k*_*12T*_ and *k*_*21T*_ are the rate constants describing the distribution of T cells between the central compartment and the tumor. *k*_*elT*_ is the elimination rate of the T cells from the central compartment. These parameters were determined from modeling of in-house PBMC data in tumor-bearing mice (not shown here). A lag time of 5 days was introduced to accommodate the disposition and start of the proliferation of T cells at the tumor site. This was informed from in-house immunohistochemistry data and was equivalent to the time of T cell observation in the tumor. *Tcells*_*tm*_ are T cells which have migrated from the central compartment to TME during the 5 days.

2. Modeling of T cell proliferation and trimer formation in the TME

**Table I:** Model Variables and Terms Used in Equations

Variable	Definition	Unit
*TotPcad*_*t*_	Total Pcad in the tumor	nM
*TotCD3*_*p*_	Total CD3 in the central compartment	nM
*TotCD3*_*t*_	Total CD3 in the tumor	nM
*Tcellsp*	T cells in the central compartment	Cells/L
*Tcellstm*	T cells migrated from plasma to tumor, during 5-day lag time	Cells/L
*Tcellst*	T cells in the tumor	Cells/L
*Dose*	Dose of PF-06671008	nmols
*C1*	Concentration of PF-06671008 in central compartment	nM
*C2*	Concentration of PF-06671008 in the peripheral compartment	nM
*C3*	Concentration of PF-06671008 in the tumor	nM
*DCD3p*	Dimer of PF-06671008-CD3 in the central compartment	nM
*DCD3t*	Dimer of PF-06671008-CD3 in the tumor	nM
*DPcadp*	Dimer of PF-06671008-Pcad in the central compartment	nM
*DPcadt*	Dimer of PF-06671008-Pcad in the tumor	nM
*Trimer*	Trimer of PF-06771008-CD3-Pcad in the tumor	nM
*w*	Total tumor volume	mm^3^
*M*_*1*_, *M*_*2*_, *M*_*3*_, *M*_*4*_	Tumor volume in growth and three transduction compartments	nM

**Table II:** Mouse Model Parameters

	**Parameter**	**Definition**	**Unit**	**Value (CV%)**			**Source**
Binding	*k*_*onCD3*_, *k*_*offCD3*_, *K*_*d_CD3*_	Binding of PF-06671008 to CD3	1/nM/h, 1/h nM	1.72, 19.66 11.4			(7)
	*k*_*onPcad*_*, k*_*offPcad*_, *K*_*d_Pcad*_	Binding of PF-06671008 to P-cadherin		1.57, 0.74 0.47			
Central/peripheral compartment	*V*_*1*_	Volume of distribution in the central compartment	mL/kg	49.6 (9)			Estimated from mouse PK data. *kel* = CL/V1 *k12* = CL_d_/V1 *k21* = Cl_d_/V2
	*V*_*2*_	Volume of distribution in the peripheral compartment	mL/kg	60.7 (16)			
	*CL*	Clearance	mL/h/kg	0.45 (12)			
	*CL*_*d*_	Inter-compartmental clearance	mL/h/kg	4.95 (28)			
	*Omega CL*	Inter individual variability in clearance	–	0.064 (41)			
	*a*	Additive error	–	0.067 (32)			
	*b*	Proportional error	–	0.207 (15)			
	*Tcells*_*p*_ *0*	Number of T cells administered/mouse	Cells/L	0, 2.5e8, 5e8, 1e9, 1.25e9, 2.5e9			See “Methods” section
	CD3	CD3 expression on T cells	Receptors/cell	100,000			(17,18)
Tumor disposition of PF-06671008/T cells	*P*	Permeability of drug into tumor	μm/day	334			(19)
	*D*	Diffusivity of drug into tumor	cm^2^/day	0.022			
	ε	Void fraction in tumor for drug	–	0.24			
	*Rcap*	Capillary radius	μm	8			
	*Rkrogh*	Average distance between 2 capillaries	μm	75			
	*Tlag*	Lag time for T cell disposition/onset of T cell proliferation in the tumor	Day	5			Set empirically, using in-house data
	*k*_*elT*_, *k*_*12T*_, *k*_*21T*_	T cell redistribution from the central compartment to the tumor	1/day	2.51, 0.002, 0.0005			
Tumor compartment	*P*_*rate*_	T cell proliferation rate	1/h	Function of dose. Eq. (9)			See “Methods” section
	*k*_*_exhaust*_^*c*^	Slope of T cell decline	1/h	0.0412			Interpolated from (20)
	*Tumor*_*cellst*_	Number of tumor cells	Cells/g of tumor	1e8			(21)
	*mPcad*	P-cadherin expression on tumor cells	Receptors/cell	28,706 (HCT-116) 17,500 (SUM-149)			(15)
	*Rtumor*	Tumor radius	cm	Calculated from w			Measured
	*k*_*int*_	P-cadherin internalization rate	Day^−1^	0.1728 (−)			Estimated from mouse tumor PK data. Represents 96 h half-life of internalization.
Tumor growth inhibition		**Mouse tumor models**		**HCT116** ^***a***^	**HCT116** ^***b***^	**SUM149** ^***b***^	
	*k*_*g*0_	Exponential tumor growth rate	1/day	0.30 (−)	0.19 (3)	0.12 (3)	Estimated in mouse models from unperturbed tumor growth data
	*k*_*g*_	Linear tumor growth rate	mm^3^/day	105 (4)	123 (2)	74.3 (5)	
	*M*_*max*_	Maximum tumor volume	mm^3^	3.8 × 10^3^ (−)	6.0 × 10^3^ (−)	5.8 × 10^3^ (−)	
	*ψ*	Switch between exponential and linear growth phases	–	20 (−)			Fixed based on (22)
	*k*_*max*_	Maximum killing rate	1/day	0.74 (7)	1.32 (7)	2.71 (14)	Estimated in mouse models
	*kC*_50_	Concentration at half maximum kill rate	nM	1.0 × 10^−4^ (6)	6.9 × 10^−5^ (7)	2.0 × 10^−4^ (15)	
	*τ*	Transduction time between tumor compartments	Day	4.78 (10)	3.99 (1)	2.25 (3)	
	*Omega k*_*g0*_	Inter-individual variability in exponential growth rate		0.46 (14)	0.34 (11)	0.12 (25)	
	*Omega k*_*g*_	Inter-individual variability in linear growth rate		0.35 (13)	0.16 (13)	0.16 (28)	
	a	Additive error		5 (−)	60 (−)	60 (−)	
	b	Proportional error		0.26 (3)	0.06 (6)	0.01(50)	
	*TSC* (*pM*) (80% confidence interval)	Tumor static concentration of the trimer		0.064 (0.044, 0.096)	0.011 (0.0096, 0.013)	0.0092 (0.0071, 0.012)	

^*a*^T cell engrafted tumor model

^*b*^T cell adoptive transfer tumor model

^*c*^Onset of exhaustion of T cells set to 7 days after disposition in the tumor

MWt of PF-06671008 = 105 kDa, MWt of sPcad = 85 kDa

T cell kinetics in the tumor: CD3+ cells/mg tumor were measured in HCT-116 tumor-bearing mice, engrafted with human PBMCs, following administration of PF-06671008 at 0.01, 0.05, or 0.5 mg/kg (described above). This data was used to determine the proliferation rate of T cells in the tumor. The relationship between CD3+ cells/mg tumor with time at each dose level was described using an exponential function. The slope of each line represents the rate of proliferation of CD3+ cells and was plotted *versus* PF-06671008 dose. An empirical model was then used to describe the CD3+ proliferation rates (*Prate*) as a function of dose (Eq. 9). Please see Supplementary Material for additional information and plots. T cells migrating into the TME during the 5-day lag time (*Tcells*_*tm*_) undergo proliferation for 7 days (Eq. 10). Following proliferation, T cells undergo contraction which was characterized using mono-exponential decline (*k*_*_exhaust*_) (Eq. 11). The time (7 days) and the rate of decline (0.0412 1/h) were estimated from literature data (20). It was assumed that T cell proliferation was only taking place in the tumor environment and that proliferation and contraction rates were the same in the human T cell engraftment and adoptive transfer mouse tumor models.

- Trimer formation: In the TME, PF-06671008 can bind to P-cadherin on tumor cells or to CD3 on T cells to form dimers, or both tumor cells and T cells to form the active trimers. The binding constants between drug and P-cadherin are *k*_*onPcad*_ and *k*_*offPcad*_ and the binding constants between drug and CD3 are *k*_*onCD3*_ and *k*_*offCD3*_. In addition to binding affinity values, trimer formation was a function of P-cadherin receptors per tumor cell (*mPcad*), number of tumor cells (*Tumor*_*cellst*_), CD3 receptors per T cell (*CD3*), and number of T cells in the TME (*Tcellst*). These values were used to calculate total Pcad (*TotPcad*_*t*_) and total CD3 (*TotCD3*_*t*_) in the TME (Eqs. 12 and 13). P-cadherin receptor expression in HCT-116 and SUM-149 tumor cell lines was determined by phycoerythrin (PE) labeling of anti-P-cadherin mAb and flow cytometry to determine the number of PE-labeled antibodies bound per cell. This study has been described previously (15). Internalization rate of drug bound to P-cadherin (*k*_*int*_) in the tumor was determined from the mouse PK study, completed in the presence of PBMCs. The number of tumor cells was determined from xenograft data. The number of CD3 receptors/T cell was taken from published data (17,18). See Eqs. 14–16 for binding of PF-06671008 to P-cadherin and CD3 to form dimers and trimers.

3. Tumor growth inhibition: The mouse xenograft PK/PD relationship was established by relating mouse PF-06671008 trimer concentration in the TME to measured xenograft tumor volume data using an optimized cell distribution transduction model (27). The presented model is a modified version of the model by Simeoni *et al.* (22). Briefly, the unperturbed tumor growth was fitted first using individual animal growth data from the vehicle control group, using a logistic model describing linear (*k*_*g*_) and exponential (*k*_*g0*_) growth. The measured initial tumor volume in each animal was used to inform the initial conditions (*M1*). M1–M4 are the tumor volume in the growth compartment and three transduction compartments, respectively. *w* is the total tumor volume (mm^3^). The inter-individual variability of the growth parameters and the maximum tumor volume (*M*_*max*_) obtained from the unperturbed growth model were then fixed in the simultaneous estimation of growth and drug effect parameters from the complete tumor volume data set. Tumor cell killing was driven by the concentration of the trimolecular complex (*Trimer*). *τ* is the transduction time, *k*_*max*_ is the maximum kill rate, *kc*_*50*_ is the concentration of the trimer in the tumor at half the maximal kill rate, and ψ is the constant for switching from exponential to linear growth patterns. Equations 17–22 describe the tumor growth inhibition modeling.

- Determination of tumor static concentration (TSC): TSC is the concentration of trimers at which tumor growth and death rate are equal, and is defined as the minimal efficacious concentration (Ceff). This PK/PD-derived parameter combines growth information and drug effect, providing insight into the efficacy of PF-06671008 in mouse xenograft models. TSC was used as a translational factor for extrapolation of xenograft data to the clinic. See Eq. 23 for TSC calculation.
  An 80% confidence interval on TSC was calculated using parametric bootstrap by resampling from the estimated parameters using a log-normal distribution.

1 $$ \frac{dC1\ }{dt}=- kel\times C1-k12\times C1+k21\times C2\times \frac{V2}{V1}- ko{n}_{CD3}\times C1\times \left( TotCD{3}_p- DCD{3}_p\ \right)+ kof{f}_{CD3}\times DCD{3}_p- Tumor\ Disposition\times \frac{TV}{V1};\mathrm{C}1\left(\mathrm{t}=0\right)=\mathrm{Dose}\ \mathrm{in}\ \mathrm{nmols} $$

2 $$ \frac{dC2\ }{dt}=k12\times C1\times \frac{V1}{V2}-k21\times C2;\mathrm{C}2\left(\mathrm{t}=0\right)=0 $$

3 $$ \mathrm{Tumor}\ \mathrm{Disposition}=\left(\frac{2\times P\times {R}_{cap}}{R_{krogh}^2}+\frac{6\times D}{R_{tumor}^2}\right)\times \left(C1-\frac{C3}{\varepsilon }\ \right) $$

4 $$ \frac{dC3\ }{dt}=\mathrm{Tumor}\ \mathrm{disposition}-{k}_{onPcad}\times C3\times \left(\frac{\left( TotPcadt- DPcadt- Trime r\right)}{\varepsilon}\right)+{k}_{of{f}_{Pcad}\kern0.5em }\times DPcadt-{k}_{onCD3}\times C3\times \left(\frac{\left( TotCD3t- DCD3t- Trime\mathrm{r}\right)}{\varepsilon}\right)+{k}_{of{f}_{CD3}\kern0.5em }\times DCD3t;\mathrm{C}3\left(t=0\right)=0 $$

5 $$ TotCD{3}_p=\left(\frac{\  Tcell{s}_p\times CD3}{6.023\times {10}^{23}}\right)\times \left(1\times {10}^9\right) $$

6 $$ {\displaystyle \begin{array}{c}\frac{dDCD{3}_p\ }{dt}= ko{n}_{CD3}\times C1\times \left( TotCD{3}_p- DCD{3}_p\ \right)- kof{f}_{CD3}\times DCD{3}_p; DCD3\left(t=0\right)=0\\ {}\end{array}} $$

7 $$ \frac{dTcell{s}_p}{dt}=- ke{l}_T\times Tcell{s}_p-k{12}_T\times Tcell{s}_p+k{21}_T\times Tcell{s}_{tm}\times \frac{TV}{V1}; Tcellsp\left(t=0\right)={Tcell}_p0 $$

8 $$ \frac{dTcell{s}_{tm}}{dt}=k{12}_T\times Tcell{s}_p\times \frac{V1}{TV}-k{21}_T\times Tcell{s}_{tm};\mathrm{Tcellstm}\left(\mathrm{t}=0\right)=0 $$

9 $$ {P}_{rate}=\left(\frac{0.014}{4+ dose}+1.5e-5\right)\times dose; $$

10 $$ Tcel{l}_t= Tcel{l}_{tm}\times {e}^{P_{rate}\times t}\ \mathrm{for}\ \mathrm{t}\le 7\ \mathrm{day}\mathrm{s},\mathrm{after}\ 5\ \mathrm{day}\ \mathrm{lag}\ \mathrm{t}\mathrm{ime} $$

11 $$ Tcel{l}_t=\left( Tcel{l}_{tm}\times {e}^{P_{rate}\times 7}\right)\times {e}^{-0.0412\times \left(t-7\right)}\ \mathrm{for}\ t>7\ \mathrm{days},\kern0.5em \mathrm{after}\ 5\ \mathrm{days}\ \mathrm{lag}\ \mathrm{time} $$

12 $$ {TotPcad}_t=\left(\frac{\  Tumo{r}_{cell{s}_t}\times mPcad}{6.023\times {10}^{23}}\right)\times \left(1\times {10}^9\right); $$

13 $$ TotCD{3}_t=\left(\frac{\  Tcell{s}_t\times CD3}{6.023\times {10}^{23}}\right)\times \left(1\times {10}^9\right); $$

14 $$ \frac{dDCD3t}{dt}={k}_{onCD3}\times C3\times \left(\frac{\left( TotCD3t- DCD3t- Trimer\right)}{\varepsilon}\right)-{k}_{of{\mathrm{f}}_{CD3}\kern0.5em }\times DCD3t-{k}_{onPcad}\times DCD3t\times \left(\frac{\left( TotPcadt- DPcadt- Trimer\right)}{\varepsilon}\right)+{k}_{of{f}_{Pcad}\kern0.5em }\times Trimer;\mathrm{DCD}3\mathrm{t}\left(\mathrm{t}=0\right)=0 $$

15 $$ \frac{dDPcadt}{dt}={k}_{onPcad}\times C3\times \left(\frac{\left( TotPcadt- DPcadt- Trimer\right)}{\varepsilon}\right)-{k}_{of{f}_{Pcad}\kern0.5em }\times DPcadt-{k}_{onCD3}\times DPcadt\times \left(\frac{\left( TotCD3t- DCD3t- Trimer\right)}{\varepsilon}\right)+{k}_{of{f}_{CD3}\kern0.5em }\times Trimer- kint\times DPcadt;\mathrm{DPcadt}\left(t=0\right)=0 $$

16 $$ \frac{dTrimer}{dt}=\kern0.5em {k}_{onCD3}\times DPcadt\times \left(\frac{\left( TotCD3t- DCD3t- Trimer\right)}{\varepsilon}\right)-{k}_{of{f}_{CD3}\kern0.5em }\times T\mathrm{r} imer+{k}_{onPcad}\times DCD3t\times \left(\frac{\left( TotPcadt- DPcadt- Trimer\right)}{\varepsilon}\right)-{k}_{o{ff}_{Pcad}}\times Trimer;\mathrm{Trimer}\left(t=0\right)=0 $$

17 $$ {k}_{kill}=\frac{k_{max}\times Trimer}{kc_{50}+ Trimer} $$

18 $$ \frac{d{M}_1}{dt}=\frac{k_{g0}\times \left(1-\frac{w}{M_{max}}\right)\times {M}_1}{{\left(1+{\left(\frac{k_{g0}}{k_g}\times w\right)}^{\psi}\right)}^{1/\psi }}-{k}_{kill}\times {M}_1;\kern0.75em \mathrm{M}1\left(t=0\right)=\mathrm{TV} $$

19 $$ \frac{d{M}_2}{dt}={k}_{kill}\times {M}_1-\frac{M_2}{\tau };\mathrm{M}2\left(t=0\right)=0 $$

20 $$ \frac{d{M}_3}{dt}=\frac{M_2-{M}_3}{\tau };\mathrm{M}3\left(t=0\right)=0 $$

21 $$ \frac{d{M}_4}{dt}=\frac{M_3-{M}_4}{\tau };\mathrm{M}4\left(t=0\right)=0 $$

22 $$ w={M}_1+{M}_2+{M}_3+{M}_4 $$

23 $$ TSC=\frac{k_{g0}\times {k}_{C50}}{k_{max}-{k}_{g0}}; $$

### Modeling

All modeling was performed using Monolix software v4.3.3 (Paris, France). The quality of the model fitting was assessed using the following:

1. Diagnostic plots: (a) plots of observations *versus* population/individual predictions and comparison with line of unity, (b) plots of weighted residuals *versus* time/concentration and check for systematic deviation from zero, (c) visual predictive checks of observations and predictions for all individuals at each dose level to check for goodness of fit.
2. Diagnostic criteria: (a) reasonable precision of the parameter estimates (RSE/CV%), (b) lack of correlation between model predicted parameters (< 0.95), (c) lack of shrinkage (η−) as a check for model over-parameterization (< 40%), (d) reduction in objective function values and/or Aikake and Schwarz criterion for model comparison.

### Translation of the Model to Human

#### Prediction of Human PK

Human PK parameters were predicted from cynomolgus monkey PK parameters using a two-compartmental PK model which incorporates binding to sPcad (Table III and Fig. 1a). PK parameters were scaled from monkey to a human using allometric exponents of 0.9 for clearance, 1 for the volume of distribution, and − 0.25 for absorption rate. These exponents were selected as they have been previously identified as optimal for monoclonal antibodies (28). The degradation rate of sPcad (*k*_*deg*_) was scaled from monkey to human using an exponent of − 0.25. The degradation rate of the PF-06671008-sPcad complex (*k*_*degcx*_) was assumed to be the same as PF-06671008 elimination rate.

**Table III:** Predicted Human Parameters Used in Simulations

	Parameter	Definition	Unit	Value (CV%)	Source
Binding	*k*_*onCD3*_, *k*_*offCD3*_, *K*_*d_CD3*_	Binding of PF-06671008 to CD3	1/nM/h, 1/h nM	1.72, 19.66, 11.4	(7)
	*k*_*onPcad*_, *k*_*offPcad*_, *K*_*d_Pcad*_	Binding of PF-06671008 to P-cadherin		1.57, 0.74, 0.47	
Central/peripheral compartment	*V*_*1*_	Volume of distribution in central compartment	mL/kg	40.2	Allometrically scaled from cynomolgus monkey PK analysis (16) *kel* = CL/V1 *k12* = CL_d_/V1 *k21* = Cl_d_/V2
	*V*_*2*_	Volume of distribution in peripheral compartment	mL/kg	211	
	*CL*	Clearance	mL/h/kg	4.61	
	*CL*_*D*_	Inter-compartmental clearance	mL/h/kg	25.2	
	*sPcad*	sPcadherin concentration in central compartment	nM	1.1 (0.4–4.1)	Measured in-house (= 92.7 ng/mL) Median value of healthy subjects and patient data
	*k*_*deg*_	sPcad degradation rate	1/h	0.15	Allometrically scaled from cynomolgus monkey PK analysis (0.31 1/h in cyno) (16)
	*k*_*degcx*_	sPcad-PF-06671008 complex degradation rate	1/h	0.115	Assumed to equal PF-06671008 elimination rate (CL/V1)
	*Tcells*_*p*_	T cell concentration in central compartment	Cells/μL	5000	(23)
	*CD3*	CD3 expression on T cells	Receptors/cell	100,000	(17,18)
Tumor disposition of PF-06671008/T cells	*P*	Permeability of drug into tumor	μm/d	334	(19)
	*D*	Diffusivity of drug into tumor	cm^2^/d	0.022	
	ε	Void fraction in tumor for drug	–	0.24	
	*Rcap*	Capillary radius	μm	8	
	*Rkrogh*	Average distance between 2 capillaries	μm	75	
	*Tcells*_*t*_^*a*^	Number of T cells in tumor	Cells/g of tumor	6.49e5	(24)
	*Tumor*_*cellst*_	Number of Tumor cells	Cells/g of tumor	1e8	(21)
	*mPcad*	P-cadherin expression on tumor cells	Receptors/cell	28,706	(15)
	*Rtumor*	Tumor radius	cm	1	Assumed
	*k*_*int*_	Internalization rate with PBMCs	Day^−1^	0.1728 (−)	Estimated from mouse tumor PK data. Represents 96 h half-life of internalization.

^*a*^Assume no proliferation in tumor; ksyn = kdeg*sPcad. MWt of PF-06671008 = 105 kDa, MWt of sPcad = 85 kDa

#### Prediction of Clinical PK/PD

The QSP model used to describe the PK/PD relationship in the mouse was translated to a human using the physiological parameters and assumptions described in Table III. An important difference from the mouse model is that PF-06671008 binds to circulating target (*sPcad*) to form drug-P-cadherin (*DPcadp*) dimers in the central compartment in the human model. The additional model equations are shown in Eqs. 24–26. In addition, T cell proliferation/contraction kinetics were not included in the human model. Instead, a “steady-state” number of T cells in the tumor are assumed (*T*_*cellst*_). All model simulations were completed using Berkeley-Madonna v8.3.18.

24 $$ \frac{dC1\ }{dt}=- kel\times C1-k12\times C1+k21\times C2\times \frac{V2}{V1}- ko{n}_{CD3}\times C1\times \left( TotCD{3}_p- DCD{3}_p\ \right)+ kof{f}_{CD3}\times DCD{3}_p- ko{n}_{Pcad}\times C1\times sPcad+ kof{f}_{Pcad}\times DPca{d}_p-\mathrm{Tumor}\ \mathrm{Disposition}\times \frac{TV}{V1};\mathrm{C}1\left(\mathrm{t}=0\right)=\mathrm{Dose}\ \mathrm{in}\ \mathrm{nmols} $$

25 $$ \frac{dsPcad\ }{dt}= ksyn- kdeg\times sPcad- ko{n}_{Pcad}\times C1\times sPcad+ kof{f}_{Pcad}\times DPca{d}_p;\mathrm{sPcad}\ \left(\mathrm{t}=0\right)=\mathrm{sPcad}\ \mathrm{in}\ \mathrm{nM} $$

26 $$ \frac{dDPca{d}_p}{dt}=\left( ko{n}_{Pcad}\times C1\times sPcad- kof{f}_{Pcad}\times DPca{d}_p\right)-{k}_{degcx}\times DPca{d}_p;\mathrm{DPcadp}\left(\mathrm{t}=0\right)=0 $$

#### Sensitivity Analyses

Local sensitivity analyses were performed to assess the sensitivity of the QSP model to P-cadherin receptor expression on tumor cells, and to tumor T cell (effector) to tumor cell ratio (E:T), as these are potentially variable parameters in patients. P-cadherin receptor numbers of 1000, 3000, 10,000, and 28,706 were used for simulations with the human model. These values represented the range of P-cadherin expression measured across human tumor cell lines (15). The nominal value of E:T used in the model was 1:150, which is thought to be representative of a solid tumor (21,24). In the sensitivity analysis, E:T ratios of tenfold lower (1:15) and tenfold higher (1:1500) than the nominal value were investigated in the human model. For quantitative comparison, sensitivity was represented as predicted tumor trimer concentration at each expression level, or E:T ratio, following an IV dose of 0.1 μg/kg PF-06671008 QW to cancer patients.

## Results

### Serum and Tumor PK of PF-06671008 in Mouse

**Fig. 2:**
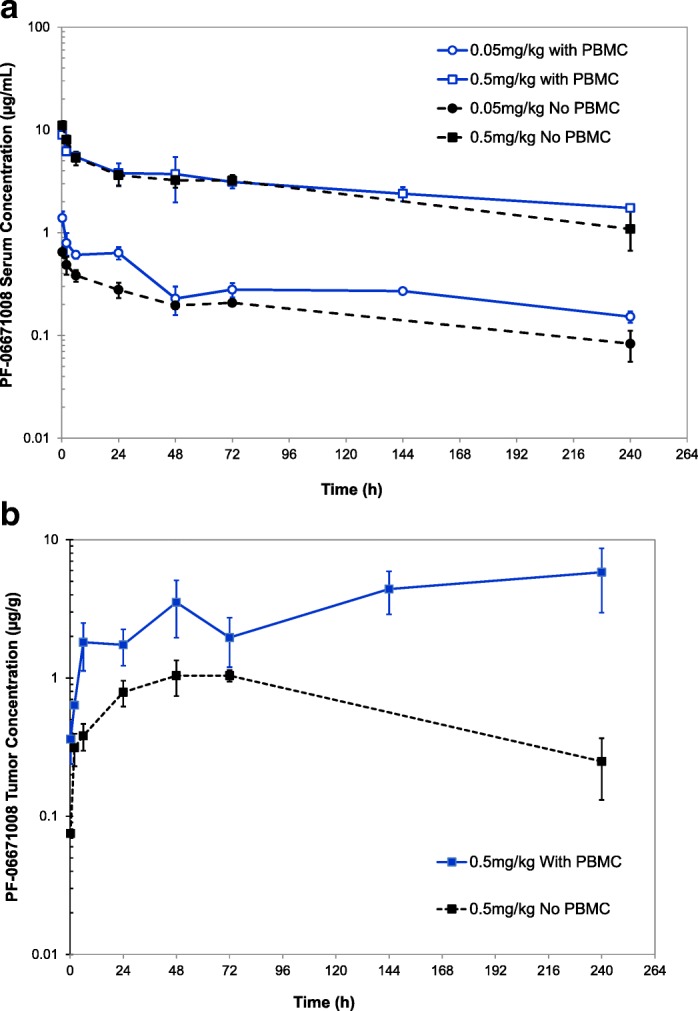
**a** Serum and **b** tumor PK profiles of PF-06671008 in PBMC engrafted and non-PBMC engrafted HCT-116 tumor-bearing mice following single-dose intravenous administration at 0.05 and 0.5 mg/kg

PK profiles of PF-06671008 in PBMC engrafted and non-PBMC engrafted HCT-116 tumor-bearing mice following single-dose IV administration at 0.05 and 0.5 mg/kg are shown in Fig. 2a. The area under the curve (AUC) of PF-06671008 in serum was dose proportional between 0.05 and 0.5 mg/kg and similar between PBMC engrafted and non-PBMC-engrafted mice (Supplemental Fig. 1). In contrast, the tumor AUC from the study with PBMC engraftment was more than fivefold higher than the study without PBMCs (Fig. 2b). This was attributed to a reduction in the internalization of PF-06671008 bound to P-cadherin on tumor cells in the presence of PBMCs.

The serum PK in the PBMC-engrafted mice was used for PK modeling. The estimated serum PK parameter estimates for PF-06671008 are shown in Table II, and the goodness of fit plots are shown in Supplemental Fig. 1. The tumor internalization rate in the presence of PBMCs was used in the TGI PK/PD modeling (Table II).

### Tumor T Cell Kinetics

**Fig. 3:**
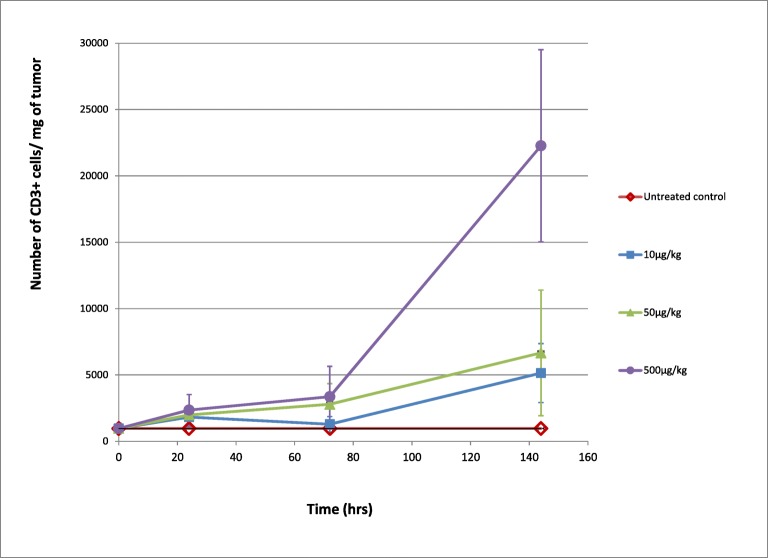
PF-06671008-induced tumor T cell proliferation in mice bearing HCT-116 tumors with human PBMC engraftment. Number of CD3+ cells/mg of the tumor (with standard deviations) is plotted against time following IV administration of control and PF-06671008 at 10 μg/kg, 50 μg/kg, and 500 μg/kg

HCT-116 tumor-bearing mice engrafted with PBMCs and administered PF-06671008 showed dose-dependent increases of tumor-infiltrating/proliferating CD3+ lymphocytes (TILs) over time (Fig. 3). The relationship with time was transformed to calculate a proliferation rate of CD3+ cells as a function of the dose which was used to describe tumor T cell kinetics in the QSP model (Supplementary Fig. 2).

### PK/PD Relationship of PF-06671008 in Mouse Xenograft Models

The QSP model (Fig. 1a) was used to fit the tumor growth inhibition data obtained from the HCT-116 and SUM-149 mouse xenograft studies. The tumor trimer concentration was used as a driver of tumor cell killing. Estimated model parameters with percent coefficient of variation (CV) and calculated tumor trimer TSCs with 80% confidence intervals are shown in Table II. Parameters were estimated with good precision as assessed by the percentage CV for all cell lines. The goodness of fit and model performance were assessed using the goodness of fit plots (population prediction, individual prediction, and visual predictive check) that are shown in Supplemental Fig. 3 for HCT-116 in the T cell engrafted model and for HCT-116 and SUM-149 in T cell adoptive transfer experimental model. Overall, the median response and variability of all cell lines were described well by the mechanistic model. The calculated population median TSCs were 0.064, 0.011, and 0.0092 pM for HCT-116 in T cell engrafted model and for HCT-116 and SUM-149 in T cell adoptive transfer experimental models, respectively. The Ceff for tumor stasis is defined as the geometric mean of the TSCs in three mouse xenograft models and was calculated to be 0.028 pM trimer concentration in the tumor.

### Serum P-Cadherin Concentrations Across Species

The concentrations of sPcad in serum samples from cynomolgus monkey, healthy humans, and cancer patients are shown in Table IV. There was no difference in sPcad levels in the serum of healthy human volunteers and cancer patients. Higher variability was observed in the lung and colorectal cancer samples compared to samples from breast cancer patients or healthy humans. Levels of sPcad in cynomolgus monkeys were similar to those in human.

**Table IV:** Concentration of Soluble P-Cadherin in Cynomolgus Monkey and Human Serum

Species	Disease state	*n*	Soluble P-cadherin concentration	
			Median (ng/mL)	Range (ng/mL)
Cynomolgus monkey^*a*^	Healthy	32	47	29–273
Cynomolgus monkey^*b*^	Healthy	4	68	57–74
Human^*a*^	Healthy	40	90	45–150
Human^*a*^	Breast cancer patients	23	78	32–190
Human^*a*^	Colon cancer patients	31	102	36–328
Human^*a*^	Lung cancer patients	25	102	65–320

^*a*^Samples from Bioreclamation (Westbury, NY)

^*b*^Samples from in-house studies

### Clinical PK Predictions for PF-06671008

The predicted human PK parameters for PF-06671008 are shown in Table III. The predicted human CL and Vss were 4.6 mL/h/kg and 251 mL/kg, respectively, and the terminal half-life was predicted to be approximately 1 day.

### Clinical PK/PD Predictions for PF-06671008 and Sensitivity to P-Cadherin Expression on Tumor Cells and T Cell Number

**Fig. 4:**
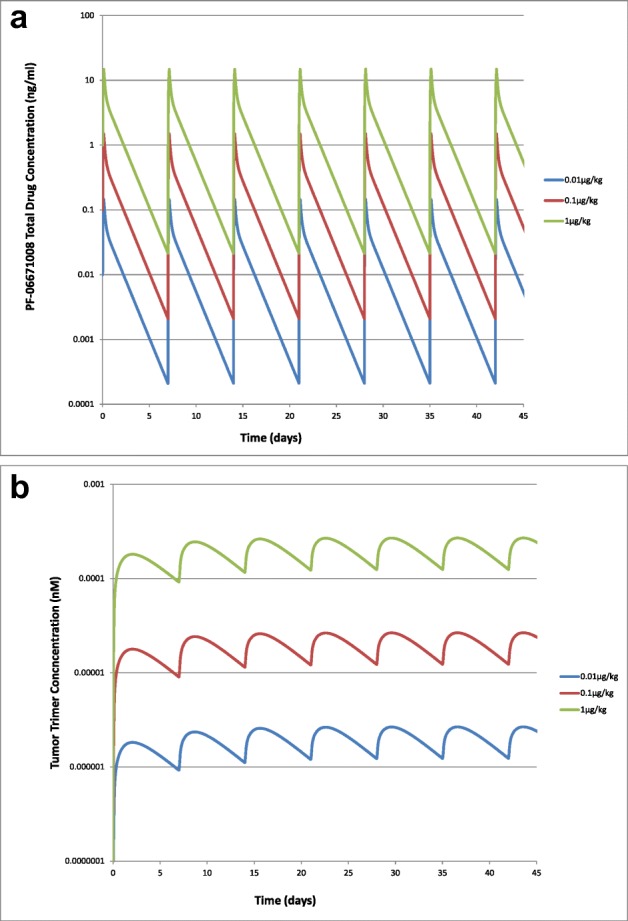
Model simulated **a** serum PK and **b** tumor trimer concentrations following IV infusion of PF-06671008 at 0.01, 0.1, and 1 μg/kg QW to cancer patients

**Fig. 5:**
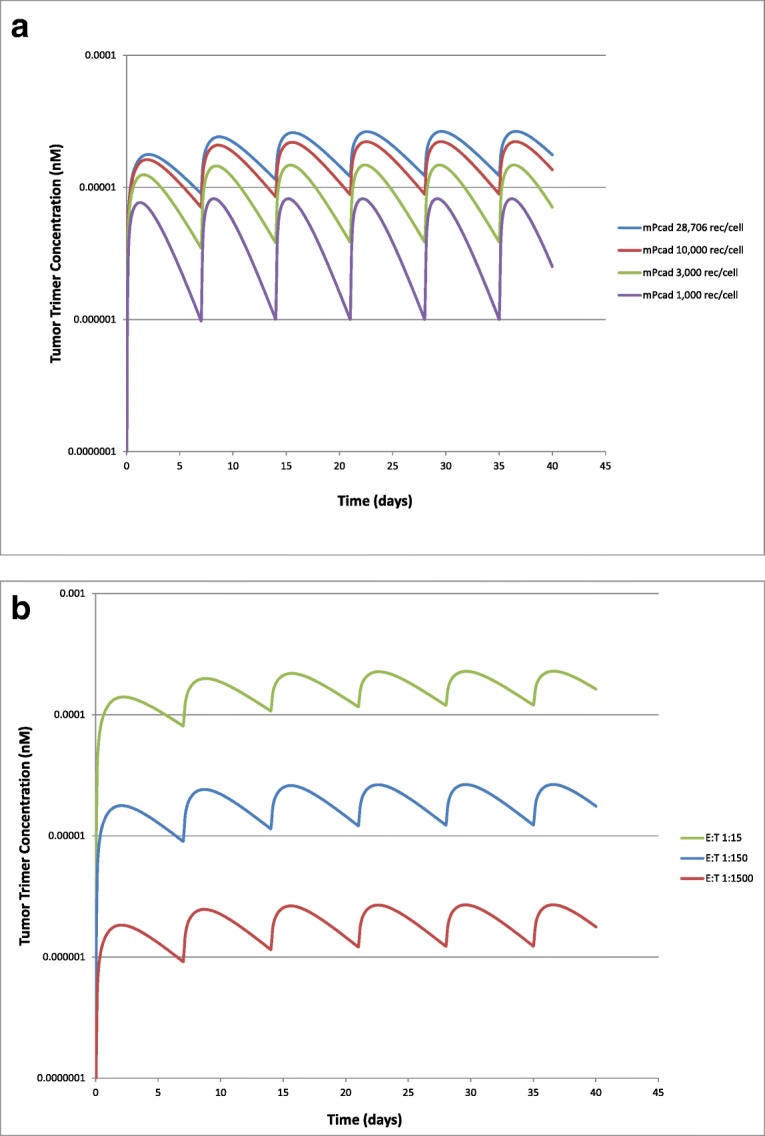
Model simulated tumor trimer concentrations at **a** different P-cadherin receptor expression values (1000–28,706 receptors/cell) and **b** different E:T ratios (1:1500–1:15) following IV infusion of PF-06671008 at 0.1 μg/kg QW to cancer patients

To translate the QSP model from mouse to human, the predicted human PK was incorporated along with assumptions and parameters describing the human physiology (Table III). The model simulated serum PK and tumor trimer concentrations following IV infusion of PF-06671008 at 0.01, 0.1, and 1 μg/kg QW to cancer patients are shown in Fig. 4 a and b, respectively. Expression levels of P-cadherin on tumor cells are expected to vary across patients. To investigate the potential impact on tumor trimer concentrations, a sensitivity analysis was performed varying P-cadherin receptor numbers from 1000 to 28,706 (HCT-116). Predicted tumor trimer concentration increases with increasing receptor expression (Fig. 5a) suggesting that P-cadherin expression is a sensitive parameter. Tumor immune status is also likely to vary across patients. The nominal E:T ratio in the model is assumed to be low (1:150) in a solid tumor (21,24). To investigate the potential impact of tumor T cell number on tumor trimer concentrations, a sensitivity analysis was performed varying E:T ratio from 1:15 to 1:1500 and assuming a constant number of tumor cells. Predicted tumor trimer concentration correlates with E:T ratio (Fig. 5b), suggesting T cells in the tumor is a sensitive parameter.

## Discussion

### Complex Exposure-Response Relationships for CD3 Bispecific Molecules

Bispecific antibodies are emerging as a leading class of biotherapeutic drugs in oncology, with the potential to enhance efficacy, increase tumor targeting and reduce systemic toxicity compared to their monospecific counterparts. These formats can vary in their molecular weight, PK, and ability to support immune effector functions. Perhaps more significantly, they can also vary in geometry a number of antigen binding sites, and the intrinsic affinity of individual arms (29). As a result of this complexity, dose-response relationships for bispecific antibodies can be non-intuitive and difficult to rationalize.

An additional complexity emerges for the CD3 bispecific T cell retargeting modality, where efficacy is driven by the formation of a trimer between the drug, T cell, and tumor cell. A bell-shaped concentration-response relationship can be observed (Fig. 1b), which is a well-described phenomenon for ternary complexes (30–33). When concentrations of antibodies are low, conditions favor the formation of trimers, with an optimal antibody concentration needed for trimer formation. However, as concentrations increase further, antibodies will be in excess and the equilibrium will shift to the formation of dimers between antibodies and T cell, or antibodies and tumor cell. This results in a decrease of response as dimers cannot trigger cytotoxicity. Since trimer concentration is a function of drug Kd values, tumor antigen expression, CD3 expression, and E:T ratio, a single drug concentration could potentially result in different trimer concentrations. Therefore, interpretation of response by drug exposure alone can be misleading. For the CD3 bispecific molecule discussed in this manuscript (PF-06671008), a bell-shaped dose-response relationship was not observed in mouse xenograft studies. This is probably because there was high P-cadherin expression on the tumor cell lines studied and good infiltration of T cells. In addition, PF-06671008 is a potent drug with low-Kd values for P-cadherin and CD3. As a result sufficient, trimer concentrations were achieved at each dose for efficacy. The bell-shaped relationship has been confirmed for other CD3 bispecific molecules in-house, where target expression is lower and/or affinity is weaker. It has also been observed in the literature from modeling of *in vitro* and *in vivo* experimental data (34,35).

### Translational QSP Model for CD3 Bispecific Molecules

QSP models, which map out the causal path between drug administration and effect in a mechanistic framework, can be a useful tool to deconvolute complex mechanisms (36). Some examples of the use of mechanistic PK/PD models to quantify and understand the system dynamics of CD3 bispecific molecules are emerging in the literature. For example, Jiang *et al.* (34) proposed a cell-killing model based on target cell-biologic-effector cell complex formation and used it to describe and predict *in vitro* cytotoxicity data for multiple T cell redirecting bispecific antibodies under different experimental conditions. Campagne *et al*. (37) developed a PK/PD model for a bispecific CD123/CD3 DART molecule in non-human primates. The model describes DART molecule binding to peripheral CD3 expressing cells and CD123+ cells, T cell trafficking, activation, and expansion, and resulting peripheral depletion of CD123 cells.

In this manuscript, a translational QSP model is proposed for CD3 bispecific T cell retargeting molecules, capable of predicting trimer formation and linking it to tumor cell killing in *in vivo* efficacy models. In addition, the mechanistic nature of the model enables the integration of patient data/parameters and subsequent clinical predictions. The model consists of 3 parts describing the central, tumor, and effect compartments (Fig. 1a). The first part includes the bispecific antibody PK, binding to circulating T cells, and binding to the soluble target (when applicable) in the central compartment. The second part describes the distribution of the antibodies to the tumor compartment using mechanistic tumor penetration equations, and parameters calculated based on the drug’s molecular weight and tumor size (19,26,38). If the model is being used for a liquid tumor, these drug exchange tumor penetration parameters can simply be removed, as liquid tumors are assumed to provide less of a diffusion barrier than solid tumors, and equilibrium can be assumed between drug concentration in the central compartment and tumor interstitium. In the tumor compartment, the model incorporates binding of the drug to CD3 on T cells and the specific antigen on tumor cells to form inactive dimers and ultimately the active trimers. In the mouse model, a simple description of T cell expansion and contraction is included, constructed using mouse TIL data and published information. For translation of this model to human, data on T cell kinetics was not available and instead a baseline concentration of T cells was assumed with no proliferation.

In the third part of the model, the trimer concentration is used as the basis for quantifying tumor volume reduction using a tumor growth inhibition model. The model used is a transduction model describing tumor cell growth and tumor cell killing (as a function of the tumor trimer concentration). The model parameters from each mouse study can be used to calculate a secondary parameter called the TSC. This is the concentration of trimer where the tumor is neither growing nor regressing and can be considered as the minimum concentration of tumor trimer required for efficacy. The TSC is a useful parameter which can be used as a pharmacodynamic index to rank compounds, or to understand the difference in compound potency across mouse xenograft models, or as the denominator in therapeutic index calculations.

### Application of the QSP Model to Quantify PK/PD Relationship for PF-06671008 in Mouse Xenograft Models

The model was used to quantify the preclinical PK/PD relationship of a CD3 bispecific molecule targeting P-cadherin (PF-06671008). To implement the model, the first step was to collect drug and system parameters describing the mechanism of action in the mouse. To calculate trimer concentration in the tumor, receptor expression of P-cadherin was determined for the HCT-116 and SUM-149 human tumor cell lines used in the mouse xenograft experiments. P-cadherin receptor expression in both cell lines (28,706 for HCT-116 and 17,500 for SUM-149) was lower than the expression of CD3 on T cells (100,000 (17,18)). This is typical for CD3 bispecific molecules as an expression of most tumor targets is less than 100,000 and as a result, tumor antigen receptor expression can be limiting and a key driver of efficacy. This was exemplified for a carcinoembryonic antigen T cell bispecific (CEA-TCB) for the treatment of solid tumors. CEA-TCB activity was found to be strongly correlated with CEA expression, with a higher potency observed in highly CEA-expressing tumor cells, with a threshold of 10,000 CEA-binding sites/cell (39). Target affinity data was also required to calculate trimer concentration. PF-06671008 binds to P-cadherin with a Kd of 0.47 nM and CD3 with a Kd of 11.4 nM (7). Binding to the tumor target antigen is often more potent than binding to CD3 on T cells in order to target the CD3 bispecific toward the tumor and away from peripheral tissues (35). In addition, strong binding to CD3 has been shown to drive more rapid clearance of an anti-CD3/anti-CLL1 bispecific in preclinical *in vivo* models (40).

The QSP model was used to integrate the mouse PK for PF-06671008 with the TGI data and to calculate TSCs in T cell engrafted (HCT-116) and T cell adoptive transfer (HCT-116 and SUM-149) established mouse tumor models. TSC values were very similar in the adoptive transfer model for both the SUM-149 and HCT-116 tumor cell lines (0.0092 and 0.011 pM respectively, with overlapping 80% confidence intervals). In contrast, a sixfold higher TSC value was obtained in the T cell engrafted *versus* T cell adoptive transfer model with the same cell line (HCT116, 0.064pM), and the respective 80% confidence intervals do not overlap. This is probably due to differences in T cell engraftment between the two mouse tumor models. In the T cell engrafted model, the T cells are administered as freshly isolated human PBMCs, 7 days prior to drug administration. In contrast, in the adoptive transfer model, activated T cells are given 1 day post-drug treatment. There are also other factors which can result in different TSCs including initial tumor size and differences in tumor growth rates.

### Translation of the Model to the Clinic

The first step in translation to human was the prediction of the clinical PK parameters. For PF-06671008, circulating soluble target can act as a sink for the drug and reduce free drug exposure by forming complexes with PF-06671008. The reduction of free sPcad concentrations in cynomolgus monkey following dosing of PF-06671008 has been reported previously (16). The human PK of PF-06671008 was predicted from cynomolgus monkey PK using a two-compartmental PK model which incorporates binding to sPcad. Levels of sPcad were measured in healthy volunteers and in breast, colon, and lung cancer patients and the median concentration in cancer patients was used in the human model.

The next step in the clinical translation process was the incorporation of human systems parameters into the QSP model. These parameters are summarized in Table III and include T cell concentration in the circulation and tumors, tumor cell concentration, and typical tumor volumes in cancer patients. Values for all of these parameters were obtained from the literature. CD3 receptor expression was kept the same as the mouse model (which used human T cells or PBMCs). P-cadherin expression of 28,706 was used in the clinical simulations. This was the value from the HCT-116 cell line and represents a medium-high level expression of P-cadherin measured across human tumor cell lines used in *in vitro* cytotoxicity experiments (874–37,582 (15)).

**Fig. 6:**
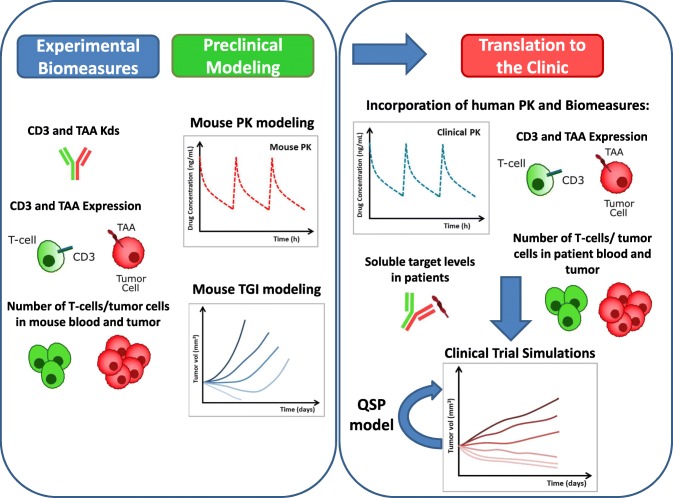
QSP model-based strategy for translating preclinical data for CD3 bispecific compounds to the clinic. “Biomeasures” can be defined as system-dependent parameters. TAA is a tumor-associated antigen

The model simulated serum PK and tumor trimer concentrations following IV infusion of PF-06671008 at 0.01, 0.1, and 1 μg/kg QW to cancer patients are shown in Fig. 4 a and b, respectively. In human, the terminal half-life of PF-06671008 was predicted to be approximately 1 day. The concentration of trimer in the tumor, which is the more relevant concentration for efficacy, accumulates slowly (Cmax approx. 2 days post first dose) and persists for longer (Fig. 4b). This is due to slow diffusion of the drug into the tumor and formation of a more stable trimer which is retained within the TME. Since receptor expression of tumor target was known to be a key parameter, a sensitivity analysis was completed using the human model with P-cadherin expression varying from 1000 to 28,706 receptors/cell. This analysis confirmed that P-cadherin receptor expression was a sensitive parameter and that concentration of trimer formed in the tumor correlates with expression level (Fig. 5a). This has an impact on predicted clinical efficacy with a higher dose required for efficacy in patients with lower P-cadherin expression. In addition, the T cell number in the tumor was found to be a sensitive parameter (Fig. 5b), with a higher predicted concentration of trimer in the tumor with increasing E:T ratio. High doses of PF-06671008 were also simulated, to check to see where the bell-shaped relationship might be observed. At doses of > 1.8 mg/kg, a reduction in tumor trimer concentration is predicted with increasing dose levels (Supplementary Fig. 4). However, at these doses, the predicted trimer concentrations in the tumor are high enough that good responses would be expected (assuming the doses would be tolerated). A translational flow diagram describing the steps taken to translate CD3 bispecific drugs from preclinical TGI data in the mouse to human is shown in Fig. 6.

The translational QSP model described for CD3 bispecific compounds can be used to drive decision-making at different stages of the drug discovery and development continuum. At early stages, the model can be used to provide guidance on the compound selection, by predicting optimal Kd values for CD3 and the tumor antigen. This can be achieved by modeling of *in vitro* data, using a reduced version of the model without the PK (central and peripheral) compartments. For example, the model was previously used to describe the *in vitro* exposure-response of PF-06671008 in cytotoxicity assays and was able to simultaneously describe the kinetics of tumor and T cells at various E:T ratios (16). Once a lead compound has been selected, the model can be used to predict clinical doses and regimens and to optimize efficient clinical study design (41). A precision medicine approach could be adopted, whereby parameters in the model such as immune cell numbers, or tumor target expression levels, are tailored to individual characteristics of patients. This could result in a recommendation of different doses for different patients. The model has also been used to predict clinical starting dose for PF-06671008 using a minimal biological effect level approach (MABEL), which is recommended for CD3 bispecific constructs due to their immune agonistic activity following target engagement (16,42). A recent analysis by the FDA concluded that receptor occupancy-based methods were not advised for CD3 bispecifics. The QSP modeling approach is more suitable to determine MABEL as efficacy is driven by drug bound to both T cells and tumor cell, rather receptor occupancy of either target singly. It is also independent of E:T ratio or other experimental specificities.

The model in its current state is very useful for a range of tasks from optimization of drug design to clinical dose predictions. However, opportunities exist to improve the model. For example, the current model includes an empirical description of T cell activation/proliferation in mouse, constructed based on TIL analysis across dose and time. A more mechanistic model could be developed by collection and characterization of more tumor lymphocyte kinetic data across species. In addition, the model is based upon a “well-mixed” hypothesis in which tumor target and T cells are assumed to be homogeneously distributed throughout the tumor environment with equal opportunity for trimer formation. However, tumors are known to be a complex environment with a heterogeneous distribution of T cells and tumor cells expressing target. Future versions of the model will take this into account.

## Conclusion

The mechanistic PK/PD model and translational framework described for CD3 bispecific molecules provide a holistic solution for quantitative decision-making throughout the drug discovery and development process. In this manuscript, the use of the model to characterize the *in vivo* PK/PD relationship of a P-cadherin/CD3 bispecific construct (PF-06671008) across mouse efficacy models is described. The model can also be translated to the clinic for human PK/PD predictions and sensitivity analysis to determine important parameters driving efficacy. The model can be applied at early stages to aid in the design of CD3 bispecific constructs and to select molecules with optimal properties.

## Electronic Supplementary Material

ESM 1 (DOCX 362 kb)

## References

[1] Farkona S, Diamandis EP, Blasutig IM (2016). Cancer immunotherapy: the beginning of the end of cancer?. BMC Med.

[2] Lameris R, de Bruin RC, Schneiders FL, van Bergenenhenegouwen PM, Verheul HM, de Gruijl TD (2014). Bispecific antibody platforms for cancer immunotherapy. Crit Rev Oncol Hematol.

[3] Baeuerle PA, Reinhardt C (2009). Bispecific T-cell engaging antibodies for cancer therapy. Cancer Res.

[4] Zimmerman Z, Maniar T, Nagorsen D (2015). Unleashing the clinical power of T cells: CD19/CD3 bi-specific T cell engager (BiTE(R)) antibody construct blinatumomab as a potential therapy. Int Immunol.

[5] Oak E, Bartlett NL (2015). Blinatumomab for the treatment of B-cell lymphoma. Expert Opin Investig Drugs.

[6] Zhu M, Wu B, Brandl C, Johnson J, Wolf A, Chow A, Doshi S (2016). Blinatumomab, a bispecific T-cell engager (BiTE((R))) for CD-19 targeted cancer immunotherapy: clinical pharmacology and its implications. Clin Pharmacokinet.

[7] Root A, Cao W, Li B, LaPan P, Meade C, Sanford J, Jin M, O’Sullivan C, Cummins E, Lambert M, Sheehan A, Ma W, Gatto S, Kerns K, Lam K, D’Antona A, Zhu L, Brady W, Benard S, King A, He T, Racie L, Arai M, Barrett D, Stochaj W, LaVallie E, Apgar J, Svenson K, Mosyak L, Yang Y, Chichili G, Liu L, Li H, Burke S, Johnson S, Alderson R, Finlay W, Lin L, Olland S, Somers W, Bonvini E, Gerber HP, May C, Moore P, Tchistiakova L, Bloom L (2016). Development of PF-06671008, a highly potent anti-P-cadherin/anti-CD3 bispecific DART molecule with extended half-life for the treatment of cancer. Antibodies.

[8] Cheung LW, Leung PC, Wong AS (2010). Cadherin switching and activation of p120 catenin signaling are mediators of gonadotropin-releasing hormone to promote tumor cell migration and invasion in ovarian cancer. Oncogene.

[9] Paredes J, Stove C, Stove V, Milanezi F, Van Marck V, Derycke L (2004). P-cadherin is up-regulated by the antiestrogen ICI 182,780 and promotes invasion of human breast cancer cells. Cancer Res.

[10] Taniuchi K, Nakagawa H, Hosokawa M, Nakamura T, Eguchi H, Ohigashi H, Ishikawa O, Katagiri T, Nakamura Y (2005). Overexpressed P-cadherin/CDH3 promotes motility of pancreatic cancer cells by interacting with p120ctn and activating rho-family GTPases. Cancer Res.

[11] Hardy RG, Tselepis C, Hoyland J, Wallis Y, Pretlow TP, Talbot I (2002). Aberrant P-cadherin expression is an early event in hyperplastic and dysplastic transformation in the colon. Gut.

[12] Imai K, Hirata S, Irie A, Senju S, Ikuta Y, Yokomine K, Harao M, Inoue M, Tsunoda T, Nakatsuru S, Nakagawa H, Nakamura Y, Baba H, Nishimura Y (2008). Identification of a novel tumor-associated antigen, cadherin 3/P-cadherin, as a possible target for immunotherapy of pancreatic, gastric, and colorectal cancers. Clin Cancer Res.

[13] Paredes J, Albergaria A, Oliveira JT, Jeronimo C, Milanezi F, Schmitt FC (2005). P-cadherin overexpression is an indicator of clinical outcome in invasive breast carcinomas and is associated with CDH3 promoter hypomethylation. Clin Cancer Res.

[14] Stefansson IM, Salvesen HB, Akslen LA (2004). Prognostic impact of alterations in P-cadherin expression and related cell adhesion markers in endometrial cancer. J Clin Oncol.

[15] Fisher TS, Hooper AT, Lucas J, Clark TH, Rohner AK, Peano B, Elliott MW, Tsaparikos K, Wang H, Golas J, Gavriil M, Haddish-Berhane N, Tchistiakova L, Gerber HP, Root AR, May C (2018). A CD3-bispecific molecule targeting P-cadherin demonstrates T cell-mediated regression of established solid tumors in mice. Cancer Immunol Immunother.

[16] Chen X, Haddish-Berhane N, Moore P, Clark T, Yang Y, Li H, Xuan D, Barton HA, Betts AM, Barletta F (2016). Mechanistic projection of first-in-human dose for bispecific immunomodulatory P-cadherin LP-DART: an integrated PK/PD modeling approach. Clin Pharmacol Ther.

[17] Carpentier B, Pierobon P, Hivroz C, Henry N (2009). T-cell artificial focal triggering tools: linking surface interactions with cell response. PLoS One.

[18] Nicolas L, Monneret G, Debard AL, Blesius A, Gutowski MC, Salles G, Bienvenu J (2001). Human gammadelta T cells express a higher TCR/CD3 complex density than alphabeta T cells. Clin Immunol.

[19] Schmidt MM, Wittrup KD (2009). A modeling analysis of the effects of molecular size and binding affinity on tumor targeting. Mol Cancer Ther.

[20] Klein L, Trautman L, Psarras S, Schnell S, Siermann A, Liblau R, Boehmer H, Khazaie K (2003). Visualizing the course of antigen-specific CD8 and CD4 T cell responses to a growing tumor. Eur J Immunol.

[21] Del Monte U (2009). Does the cell number 10(9) still really fit one gram of tumor tissue?. Cell Cycle.

[22] Simeoni M, Magni P, Cammia C, De Nicolao G, Croci V, Pesenti E (2004). Predictive pharmacokinetic-pharmacodynamic modeling of tumor growth kinetics in xenograft models after administration of anticancer agents. Cancer Res.

[23] Yarbro CH, Frogge, Margaret Hansen, Goodman, Michelle. Cancer nursing: principles and practice. 6 ed. Jones & Bartlett Learning; 2005.

[24] Kovacsovics-Bankowski M, Chisholm L, Vercellini J, Tucker CG, Montler R, Haley D, Newell P, Ma J, Tseng P, Wolf R, Vetto JT, Hammill C, Hansen P, Weinberg AD (2014). Detailed characterization of tumor infiltrating lymphocytes in two distinct human solid malignancies show phenotypic similarities. J Immunother Cancer.

[25] Thurber GM, Dane Wittrup K (2012). A mechanistic compartmental model for total antibody uptake in tumors. J Theor Biol.

[26] Thurber GM, Schmidt MM, Wittrup KD (2008). Antibody tumor penetration: transport opposed by systemic and antigen-mediated clearance. Adv Drug Deliv Rev.

[27] Haddish-Berhane N, Shah DK, Ma D, Leal M, Gerber HP, Sapra P, Barton HA, Betts AM (2013). On translation of antibody drug conjugates efficacy from mouse experimental tumors to the clinic: a PK/PD approach. J Pharmacokinet Pharmacodyn.

[28] Betts A, Keunecke A, van Steeg TJ, van der Graaf PH, Avery LB, Jones H, Berkhout J (2018). Linear pharmacokinetic parameters for monoclonal antibodies are similar within a species and across different pharmacological targets: a comparison between human, cynomolgus monkey and hFcRn Tg32 transgenic mouse using a population-modeling approach. MAbs.

[29] Mazor Y, Hansen A, Yang C, Chowdhury PS, Wang J, Stephens G, Wu H, Dall’Acqua WF (2015). Insights into the molecular basis of a bispecific antibody’s target selectivity. MAbs.

[30] Douglass EF, Miller CJ, Sparer G, Shapiro H, Spiegel DA (2013). A comprehensive mathematical model for three-body binding equilibria. J Am Chem Soc.

[31] Verhamme IM (2012). Fluorescent reporters of thrombin, heparin cofactor II, and heparin binding in a ternary complex. Anal Biochem.

[32] Lever M, Lim HS, Kruger P, Nguyen J, Trendel N, Abu-Shah E, Maini PK, van der Merwe PA, Dushek O (2016). Architecture of a minimal signaling pathway explains the T-cell response to a 1 million-fold variation in antigen affinity and dose. Proc Natl Acad Sci U S A.

[33] Duensing TD, Putten JP (1998). Vitronectin binds to the gonococcal adhesin OpaA through a glycosaminoglycan molecular bridge. Biochem J.

[34] Jiang X, Chen X, Carpenter TJ, Wang J, Zhou R, Davis HM, Heald DL, Wang W (2018). Development of a target cell-biologics-effector cell (TBE) complex-based cell killing model to characterize target cell depletion by T cell redirecting bispecific agents. MAbs..

[35] Mandikian D, Takahashi N, Lo AA, Li J, Eastham-Anderson J, Slaga D, Ho J, Hristopoulos M, Clark R, Totpal K, Lin K, Joseph SB, Dennis MS, Prabhu S, Junttila TT, Boswell CA (2018). Relative target affinities of T-cell-dependent bispecific antibodies determine biodistribution in a solid tumor mouse model. Mol Cancer Ther.

[36] Agoram BM, Martin SW, van der Graaf PH (2007). The role of mechanism-based pharmacokinetic-pharmacodynamic (PK-PD) modelling in translational research of biologics. Drug Discov Today.

[37] Campagne O, Delmas A, Fouliard S, Chenel M, Chichili GR, Li H, Alderson R, Scherrmann JM, Mager DE (2018). Integrated pharmacokinetic/pharmacodynamic model of a bispecific CD3xCD123 DART molecule in nonhuman primates: evaluation of activity and impact of immunogenicity. Clin Cancer Res.

[38] Thurber GM, Schmidt MM, Wittrup KD (2008). Factors determining antibody distribution in tumors. Trends Pharmacol Sci.

[39] Bacac M, Fauti T, Sam J, Colombetti S, Weinzierl T, Ouaret D, Bodmer W, Lehmann S, Hofer T, Hosse RJ, Moessner E, Ast O, Bruenker P, Grau-Richards S, Schaller T, Seidl A, Gerdes C, Perro M, Nicolini V, Steinhoff N, Dudal S, Neumann S, von Hirschheydt T, Jaeger C, Saro J, Karanikas V, Klein C, Umana P (2016). A novel carcinoembryonic antigen T-cell bispecific antibody (CEA TCB) for the treatment of solid tumors. Clin Cancer Res.

[40] Leong Steven R., Sukumaran Siddharth, Hristopoulos Maria, Totpal Klara, Stainton Shannon, Lu Elizabeth, Wong Alfred, Tam Lucinda, Newman Robert, Vuillemenot Brian R., Ellerman Diego, Gu Chen, Mathieu Mary, Dennis Mark S., Nguyen Allen, Zheng Bing, Zhang Crystal, Lee Genee, Chu Yu-Waye, Prell Rodney A., Lin Kedan, Laing Steven T., Polson Andrew G. (2016). An anti-CD3/anti–CLL-1 bispecific antibody for the treatment of acute myeloid leukemia. Blood.

[41] Schropp Johannes, Khot Antari, Shah Dhaval K., Koch Gilbert (2019). Target‐Mediated Drug Disposition Model for Bispecific Antibodies: Properties, Approximation, and Optimal Dosing Strategy. CPT: Pharmacometrics & Systems Pharmacology.

[42] Saber Haleh, Del Valle Pedro, Ricks Tiffany K., Leighton John K. (2017). An FDA oncology analysis of CD3 bispecific constructs and first-in-human dose selection. Regulatory Toxicology and Pharmacology.

