# Supplementary material for: A Translational Quantitative Systems Pharmacology Model for CD3 Bispecific Molecules: Application to Quantify T Cell-Mediated Tumor Cell Killing by P-Cadherin LP DART®
Source: AAPS J. 2019 May 22;21(4):66. doi: 10.1208/s12248-019-0332-z (PMC6531394; doi:10.1208/s12248-019-0332-z)
Supplement: Supplementary file 1 — (DOCX 362 kb) [file 12248_2019_332_MOESM1_ESM.docx]

**Supplemental Files:**

**Supplemental Figure 1**

Goodness of fit plot for PF-06671008 PK model fitting in PBMC engrafted HCT-116 tumor bearing mice following IV administration at 0.05mg/kg and 0.5mg/kg. The cyan band represents the 95% prediction distribution.

**
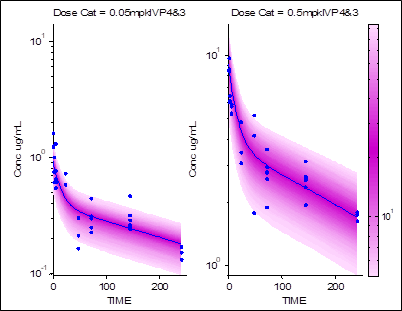
**

**Supplemental Figure 2**

CD3+ cells/ mg tumor versus time relationship following IV administration of PF-06671008 at (a) 10µg/kg, (b) 50µg/kg and (c) 500µg/kg. An exponential function was fitted to the data and the equations are shown on each plot. (d) The slope of each line is plotted versus dose, and an empirical model fitted to the data.

(a)


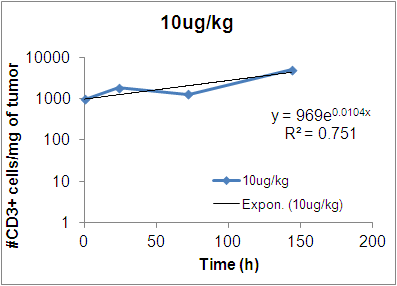


(b)


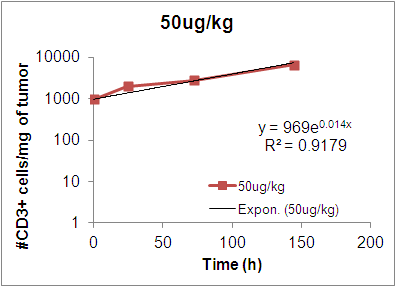


(c)

**
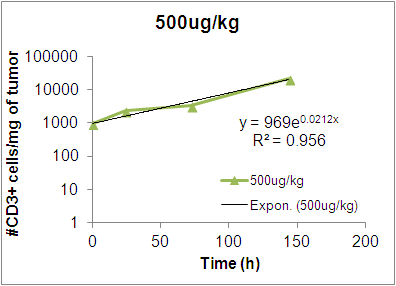
**

(d)

**
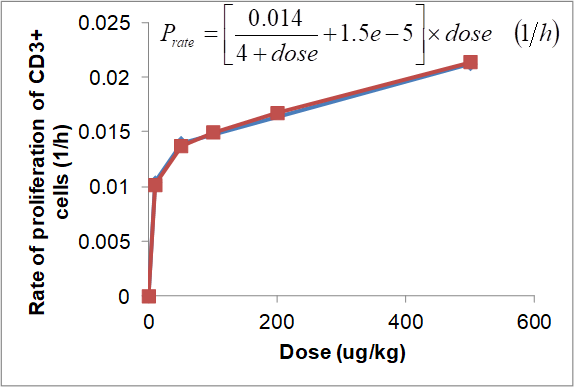
**

**Supplemental Figure 3**

Goodness of fit plots for (a) HCT-116 in T-cell engrafted model, (b) HCT-116 in T-cell adoptive transfer model, and (c) SUM-149 in T-cell adoptive transfer model.

Plots shown include: (i) observations versus model predictions using the population and individual parameters compared to line of unity and (ii) visual predictive checks of tumor volume (mm3) data and model prediction versus time at each dose level. The cyan band represents the 95% prediction distribution.

**(a)**


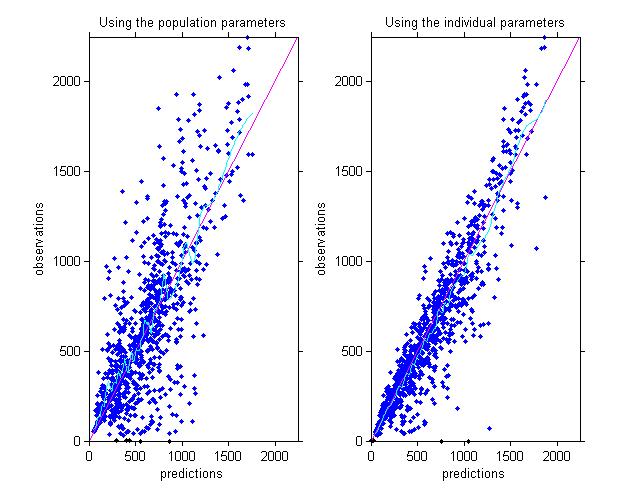

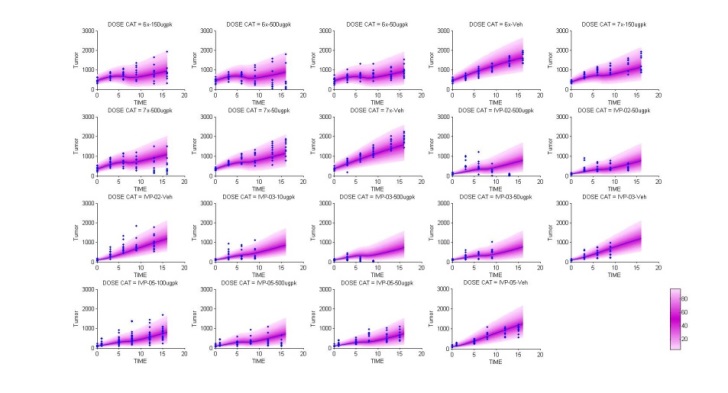


**(b)**


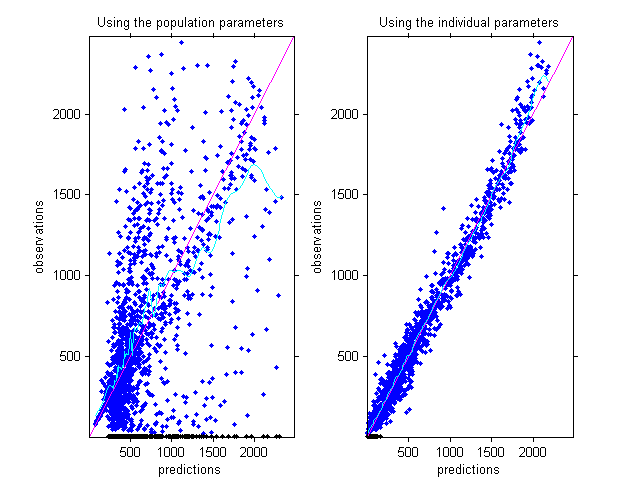

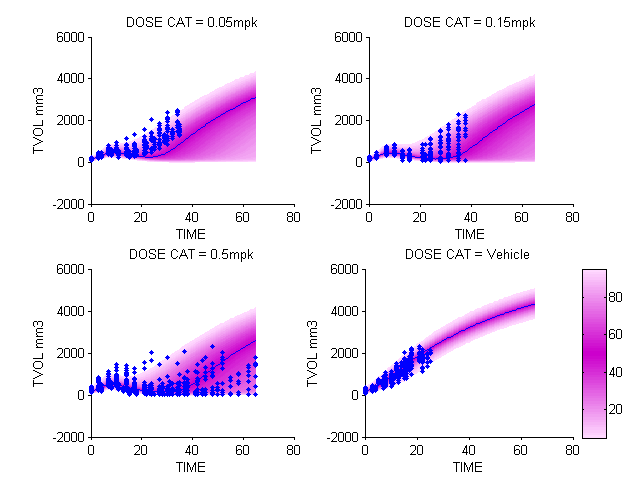


**(c)**


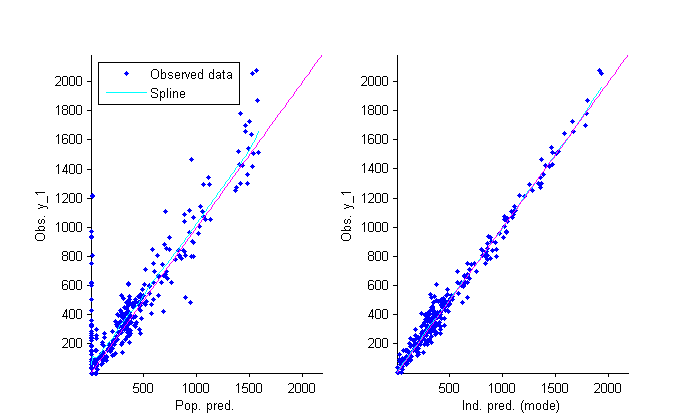

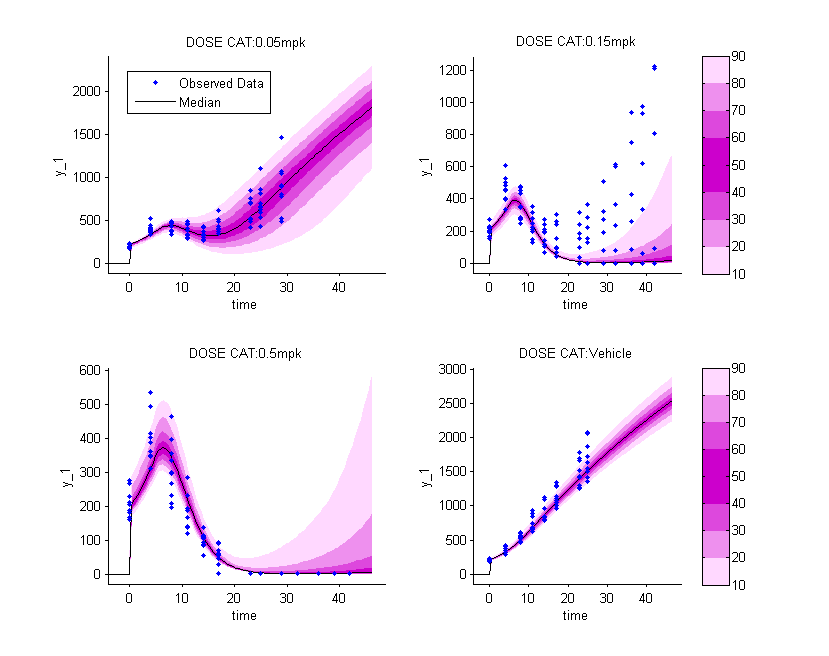


**Supplemental Figure 4**

Model simulated tumor trimer concentrations following IV infusion of PF-06671008 at 0.01, 0.06, 0.3, 1.8 and 10mg/kg QW to cancer patients. At doses of >1.8mg/kg predicted tumor trimer concentrations start to decrease with increasing dose, representing the bell shaped relationship.

**
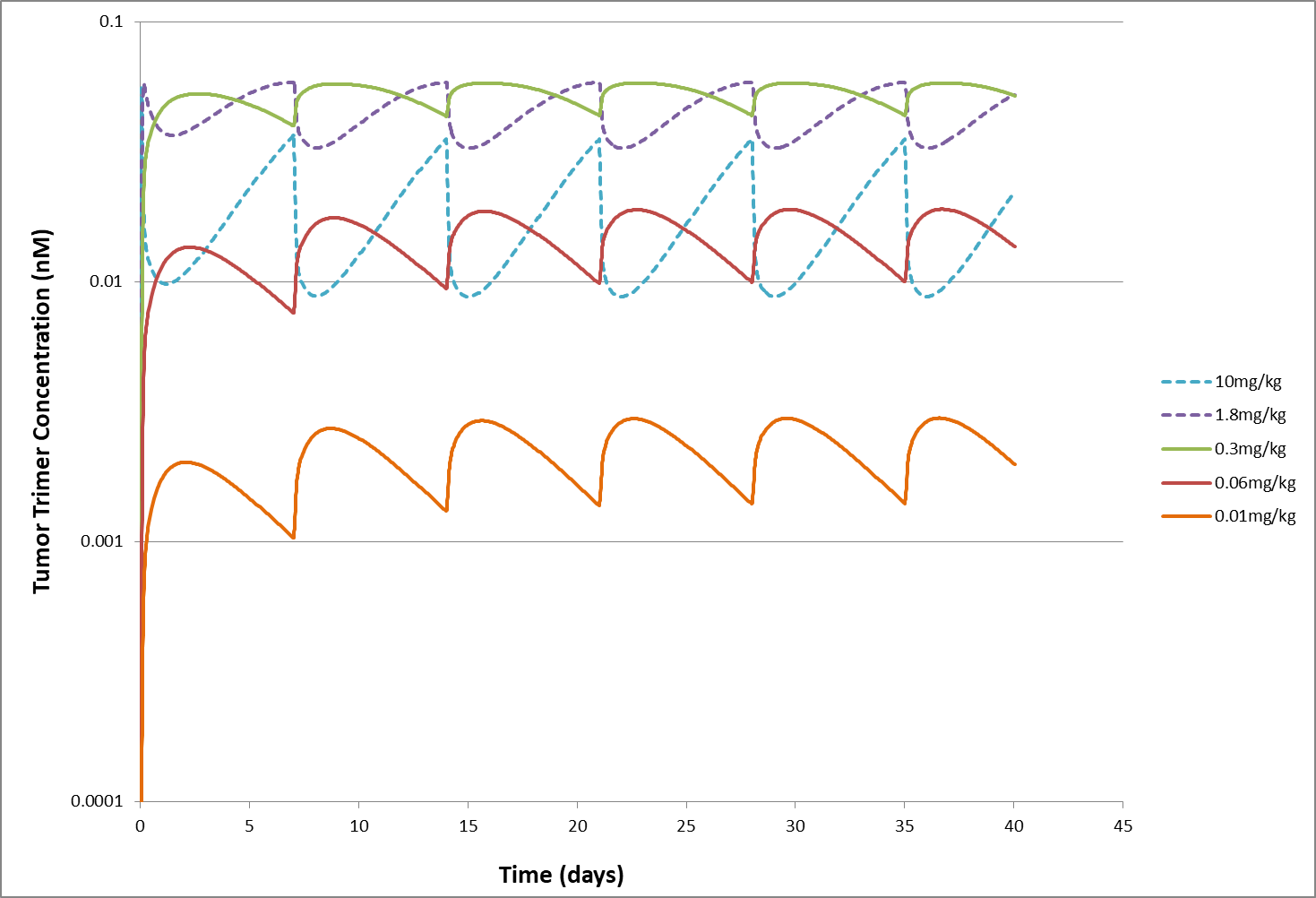
**
